# WINGLESS (WNT) signaling is a progesterone target for rat uterine stromal cell proliferation

**DOI:** 10.1530/JOE-15-0523

**Published:** 2016-05-01

**Authors:** Virginia Rider, Alex Talbott, Anuradha Bhusri, Zach Krumsick, Sierra Foster, Joshua Wormington, Bruce F Kimler

**Affiliations:** 1 Department of BiologyPittsburg State University, Pittsburg, Kansas, USA; 2 Department of Radiation OncologyThe University of Kansas Medical Center, Kansas City, Kansas, USA

**Keywords:** cell proliferation, progesterone, *Wnt* signaling, uterus

## Abstract

Preparation of mammalian uterus for embryo implantation requires a precise sequence of cell proliferation. In rodent uterus, estradiol stimulates proliferation of epithelial cells. Progesterone operates as a molecular switch and redirects proliferation to the stroma by down-regulating glycogen synthase kinase-3β (GSK-3β) and stimulating β-catenin accumulation in the periluminal stromal cells. In this study, the WNT signal involved in the progesterone-dependent proliferative switch was investigated. Transcripts of four candidate *Wnt* genes were measured in the uteri from ovariectomized (OVX) rats, progesterone-pretreated (3 days of progesterone, 2mg/daily) rats, and progesterone-pretreated rats given a single dose (0.2µg) of estradiol. The spatial distribution of the WNT proteins was determined in the uteri after the same treatments. *Wnt5a* increased in response to progesterone and the protein emerged in the periluminal stromal cells of progesterone-pretreated rat uteri. To investigate whether WNT5A was required for proliferation, uterine stromal cell lines were stimulated with progesterone (1µM) and fibroblast growth factor (FGF, 50ng/mL). Proliferating stromal cells expressed a two-fold increase in WNT5A protein at 12h post stimulation. Stimulated stromal cells were cultured with actinomycin D (25µg/mL) to inhibit new RNA synthesis. Relative *Wnt5a *expression increased at 4 and 6 h of culture, suggesting that progesterone plus FGF preferentially increased *Wnt5a* mRNA stability. Knockdown of *Wnt5a* in uterine stromal cell lines inhibited stromal cell proliferation and decreased *Wnt5a* mRNA. The results indicate that progesterone initiates and synchronizes uterine stromal cell proliferation by increasing WNT5A expression and signaling.

## Introduction

In adult female mammals, the uterus undergoes remodeling with a regular cyclicity under the control of the sex hormones, progesterone and estradiol (Bell 1983, Pawar *et al*. 2014). In the rodent uterus, estradiol stimulates the proliferation of luminal and glandular epithelial cells at days 2 and 3 *post-coitum *(Finn & Martin 1967). At day 4 of pregnancy in rats, cell division switches from the luminal and glandular epithelium to the endometrial stroma in response to progesterone (Rider & Psychoyos 1994). Stromal cells proliferate and differentiate to form the maternal tissue referred to as the decidua that interfaces with the fetal placenta. Differentiation of stromal cells is accompanied by an increase in cell size, polyploidization, and changes in gene expression including increased expression of decidual prolactin, IGF-binding protein 1 (IGFBP1) and fibroblast growth factor (FGF). Mice lacking genes that control decidualization cannot maintain pregnancy (Laws *et al.* 2008, Wang *et al.* 2013).

WNT glycoproteins comprise a family of at least 19 ligands that bind to G-protein-coupled frizzled receptors and two low-density lipoprotein receptor-related protein co-receptors (Paul & Dey 2008, Sonderegger *et al*. 2010, Tepekoy *et al*. 2015). WNT signaling is required for proper development of the mammalian female reproductive tract (Parr & McMahon 1998). Female mice that lack WNT5A by targeted deletion do not develop posterior reproductive tract structures (Mericskay *et al.* 2004). WNT5A and WNT7A are required for proper uterine gland formation (Dunlap *et al.* 2011, Hayashi *et al.* 2011). WNT4 deficiency in humans results in the insufficient formation of the Mullerian duct of the female reproductive tract (Philibert *et al*. 2008). WNT4-deficient females develop Wolffian ducts owing to the production of testosterone in mice lacking WNT4 (Vainio *et al.* 1999). 

Less is known about the integration of WNT signaling in the adult uterus, although changes in the spatial and temporal expression of *Wnt* genes during the implantation period suggest their importance in the establishment of pregnancy (Mohamed *et al.* 2005, Sonderegger *et al.* 2010, Hayashi *et al.* 2011, Fritz *et al.* 2014). The Wnt/β-catenin pathway plays a critical function at the site of implantation as inhibition of this signaling pathway interferes with the process (Mohamed *et al*. 2005). Several WNT proteins (WNT4, WNT5A, WNT6, and WNT7A) are highly expressed in the adult uterus (Hayashi *et al.* 2011, Li *et al.* 2013, Wang *et al.* 2013). Ablation of *Wnt4* in the adult mouse uterus leads to hypertrophy of the luminal epithelium and implantation failure (Franco *et al*. 2011). Inactivation of *Wnt5a *in adult mouse uteri has resulted in compromised fertility, whereas overexpression of the protein has decreased litter size (Cha *et al*. 2014). The results from the study (Cha *et al.* 2014) suggests that decidualization requires WNT5A in the appropriate amounts for decidualization and normal pregnancy. *Wnt6*-null mutant mice show a reduction in litter size (Wang *et al*. 2013). Interestingly, embryo attachment appeared normal in the absence of *Wnt6*; however, at days 6–7 of pregnancy, the weight and size of implantation sites had declined. *Wnt7*a-knockout mice do not express *Hoxa10* and *Hoxa11* in the endometrial stroma and stromal cell differentiation fails and infertility ensues (Miller & Sassoon 1998). Postnatal deletion of *Wnt7a* diminishes the formation of uterine glands. The number of embryos recovered from the uteri of the mutant mice, however, was not different from wild-type mice, indicating that implantation failed in the mutants rather than an ovulation or embryo defect (Dunlap *et al.* 2011).

Although ablation of various *Wnt* genes has established their importance in embryo–maternal interactions, *Wnt* signal transduction mechanisms in the different reproductive cell types is not well understood. In the uteri of adult mammals, female sex steroids direct changes that alter the uterus from a hostile to a receptive state for embryo implantation (Franco *et al*. 2012, Fritz *et al.* 2014, Pawar *et al.* 2014). Hormones stimulate uterine cell proliferation by a variety of mechanisms, including the induction of growth factors/growth factor receptors and paracrine signaling, and by direct regulation of cell cycle genes (Jones *et al*. 2000, Yao *et al*. 2003, Butt *et al.* 2008). In the rodent uterus, estradiol stimulates epithelial cell proliferation, whereas progesterone redirects proliferation from the epithelial to the stromal compartment. Administration of progesterone to ovariectomized (OVX) rats for three consecutive days increases the number of proliferating stromal cells approximately five-fold (Rider & Psychoyos 1994). This proliferative switch is accompanied by decreased GSK-3β expression in progesterone-pretreated uterine stromal cells and increased accumulation of β-catenin (Rider *et al.* 2006). β-catenin is a transcriptional regulator that interacts with T-cell factor (TCF/lymphoid enhancer factor (LEF)) and converts the TCF/LEF repressor complex into a transcriptional activator (Daniels & Weis 2005, Willert & Jones 2006). Progesterone stimulates the accumulation of β-catenin in the uterine stromal cells, whereas estradiol stimulates its nuclear translocation. Nuclear β-catenin increases complex formation with LEF and activates *Wnt* target genes (Rider *et al.* 2006). Targeted disruption of normal β-catenin regulation results in infertility, and the uteri in mutant mice cannot undergo the decidual reaction (Jeong *et al.* 2009).

In this study, we have extended our earlier observations in order to identify the endocrine-responsive *Wnt* gene(s) involved in stromal cell proliferation. *Wnt5a* expression increased in progesterone-pretreated rat uteri and the protein localized to the presumptive decidual cells. Stromal cell lines stimulated with progesterone and FGF exhibited an increase in cell number and a two-fold increase in WNT5A protein. Addition of actinomycin D to inhibit *Wnt5a* expression did not reduce *Wnt5a *transcripts, suggesting that progesterone plus FGF enhances expression, in part, by stabilizing *Wnt5a* mRNA. ­Knockdown of *Wnt5a* mRNA in proliferating stromal cells decreased *Wnt5a* expression and blocked progesterone-dependent proliferation.

## Materials and methods

### Animals and hormone treatments

Sexually mature (150–175 g body weight) Sprague–Dawley rats (Charles River Laboratories) were bilaterally OVX and rested for 10 days. Rats were housed on a 14 h light:1 0h darkness cycle at the Pittsburg State University and provided rodent chow and water *ad libitum*. Animals were treated in accordance with the principles and procedures outlined in the NIH Guidelines for the Care and Use of Experimental Animals. The Pittsburg State University Animal Care Committee approved protocols for the care and the use of rats. To stimulate stromal cell proliferation, OVX rats were injected subcutaneously (s.c.) with progesterone (2mg; Sigma-Aldrich) and dissolved in sesame oil daily for three consecutive days. On the fourth day, estradiol 17-β (0.2μg; Sigma-Aldrich) was injected s.c. This hormone regimen increases the number of synchronously proliferating stromal cells three- to five-fold compared with normally pregnant animals (Rider & Psychoyos 1994). The uterine horns were removed at 6 h post estradiol injection when a significant number of stromal cells are in S phase of the cell cycle (Rider *et al.* 2003*a*).

### Indirect immunoperoxidase analysis

Uterine tissue was removed under anesthesia and the uterine horns from OVX rats, those pretreated with progesterone for 72h (0 hE), and those pretreated with progesterone and estradiol for 6h (6hE) were fixed in 4% paraformaldehyde and embedded in paraffin using methods standard in our laboratory (Rider *et al.* 2006). Sections (~8μm) were cut on a microtome and placed on Superfrost Plus slides (Fisher Scientific, Hanover Park, IL, USA). Sections were treated in 10mM sodium citrate at 95°C for 5min to unmask the antigens. To remove endogenous peroxidase activity, tissue sections were quenched in 0.3% hydrogen peroxide (Sigma-Aldrich) in methanol at 22°C for 30min. Samples were blocked for 18 h in a blocking buffer (50mM Tris–HCl, pH 7.4, 150mM NaCl) containing 1% normal bovine serum. The slides were washed in phosphate-buffered saline (PBS) and reacted with WNT-specific antibodies (15μg/mL) (WNT4, AF475 R&D Systems; WNT5a AF645, R&D Systems; WNT7a AF3008, R&D Systems; WNT7b AF3460, R&D Systems). To evaluate the specificity of the reaction, some sections were incubated without primary antibody. Sections were reacted with biotinylated affinity-purified species-specific secondary antibodies (Vector Laboratories) for 60min at 22°C. Slides were reacted with the Vectastain Elite ABC reagent (Vector Laboratories), washed in PBS, and reacted for 2min with equal volumes of 1μg/mL diaminobenzidine (Aldrich, Milwaukee, WI, USA) dissolved in 0.1 M Tris–HCl, pH 7.2, and 0.1% (v/v) hydrogen peroxide diluted in PBS. Slides were counterstained with 1% methyl green dye in deionized water. The slides were mounted using Permount. The uterine horns from at least three animals at each time point were examined. There was no brown reaction product in the absence of primary antibodies. Representative sections were photographed using an Olympus BX41 microscope equipped with a digital camera.

### RNA isolation

Uterine horns were pooled from three sexually mature Sprague–Dawley rats after each treatment (OVX, 0hE, 6hE) and homogenized in 4mL TRIzol reagent using a Virtis Tempest Blade-type homogenizer at high-speed setting (setting 6). The entire cell lysate solution was added to Phase lock Gel Heavy Eppendorf tubes and incubated for 5min at 22°C. Then, chloroform (0.2mL) was added and the tubes were vigorously shaken for 15s. Samples were centrifuged at 12,000***g*** for 10min at 4°C. RNA was precipitated by adding muscle glycogen (10μg; Ambion) and 0.5mL isopropyl alcohol. RNA concentration and purity were determined by absorbance (260/280nm) using a spectrophotometer.

### Reverse transcription and polymerase chain amplification (RT-PCR)

Complementary DNA (cDNA) was synthesized with 1–4µg RNA using a High Capacity cDNA Kit (Applied Biosystems). Semiquantitative PCR amplification using a Peltier Thermocycler (MJ Research, San Francisco, CA, USA) was employed to measure *Wnt* mRNAs. The primers for glyceraldehyde phosphate dehydrogenase have been reported: *Gapdh* sense (5′-GAG TCA ACG GAT TTG GTC GT-3′); *Wnt4* sense (5′-TGT ACC TGG CCA AGC TGT CAT-3′); *Wnt5a* sense (5′-TCC TAT GAG AGC GCA CGC AT-3′); *Wnt7a *sense (5′-CAA GGC CAG TAC CAC TGG GA-3′); and *Wnt7*b sense (5′-ACC AAA ACT TGC TGG ACC AC-3′) (Mohamed *et al*. 2004). Conditions for amplification were determined empirically. Templates were denatured at 94°C 1min, annealed between 60 and 62°C 1min, and elongated at 72°C 1min for 35cycles. Annealing temperatures varied slightly (*Wnt4*=61°C, *Wnt5a*=60.5°C, *Wnt7a*=60°C, *Wnt7b*=62°C). The PCR products (20μL) were electrophoresed on 2% agarose gels. Amplified bands were photographed and the amount of *Wnt* was measured by scanning densitometry. The optical density of the target gene was divided by the optical density of *Gapdh *amplified from the same template.

### Cell culture

Isolation and characterization of the uterine stromal cell lines are described in detail elsewhere (Piva *et al.* 1996). Briefly, the stromal cell lines were isolated from sexually mature Sprague–Dawley rat uteri. The cells express mesenchymal markers and female sex steroid (estradiol and progesterone) receptors. Proliferation of stromal cells was blocked by progesterone receptor antagonism. The proliferative response to progesterone plus FGF was studied by flow cytometry (Rider *et al*. 1998) and the MTT assay (Piva *et al*. 1996). Entry into DNA replication was monitored using ^3^H thymidine (Rider *et al*. 1998) and BrdU incorporation (Rider *et al*. 2003*a*). In this study, cells from the same passage (UIII, passages 20–25) were used to determine the treatment effects within an experiment by propagating a sufficient number of cells for each experiment in medium 199 containing 10% FBS. Uterine stromal cells were seeded (1×10^4^ per well) in 24-well plates and quiescence was induced by culture for 72h in serum-free, phenol red-free Dulbecco’s Modified Eagles’ medium (Gibco) containing molecular cellular development biology (MCDB)-105 (Sigma-Aldrich) in a 3:1 mixture. The medium contained insulin (5μg/mL) and other supplements as detailed elsewhere (Piva *et al*. 1996). Quiescent cells were stimulated to synchronously enter the cell cycle by adding progesterone (1µM) and FGF (50ng/mL). These doses were previously determined to be optimal in dose–response studies (Piva *et al.* 1996). Some cells were treated with progesterone or FGF alone. Stromal cells were collected at various time points (0–48h) after addition of mitogenic agents. 

Total RNA and proteins were sequentially separated from the same stromal cell samples by column purification (Norgen Biotek, Thorold, ON, Canada). Briefly, RNA was bound to the column and the proteins were collected in the flow through. RNA was treated with DNase I and eluted from the column. The pH of the flow through was adjusted, the proteins were bound to the column, and the columns were washed. The proteins were eluted and stored at –80°C. Protein concentration was determined using the Bradford Reagent (Bio-Rad) and RNA concentration was measured by absorbance (260/280 nm) using a spectrophotometer. 

### Western blots

Uterine stromal cell extracts were heated at 95°C for 3min in SDS-sample buffer, cooled to 22°C, and the proteins were size fractioned by SDS–PAGE. Proteins were transferred to a nitrocellulose membrane using standard methods (Jones *et al*. 2000, Rider *et al.* 2003*a*). For the chemiluminescent detection, the nitrocellulose membranes were reacted with a WNT5A antibody (1µg/mL; R&D) at 22°C for 60min. Some samples were treated identically except the membranes were reacted without the primary antibody (data not shown). The membranes were washed and incubated for 60min with a species-specific horseradish peroxidase (HRP)-conjugated secondary antibody (1:50,000; Pierce Biotechnology). The blots were incubated with a SuperSignal West Femto Maximum Sensitivity Substrate (Pierce Biotechnology) for 5min. The blots were exposed on chemiluminescent film to visualize WNT5A protein. The size of the reactive proteins was determined from prestained molecular size standards (Bio-Rad). The blots were stripped and reacted with a β-actin antibody (AC-15, Sigma) to control for equal protein loading. Scanning densitometry of β-actin across treatment groups and assays indicated that the amount of protein did not vary more than 7%, verifying its lack of response to treatments (Rider *et al.* 2006). The relative amount of WNT5A was measured by scanning densitometry. The optical density of WNT5A was divided by the optical density of β-actin in the same sample. Two independent experiments were performed. 

### *Wnt5a* mRNA stability

To determine the effects on mRNA stability, stromal cells were plated (2×10^5 ^cells per dish) in serum-free medium for 72h to induce quiescence. The cells were stimulated with progesterone, FGF, and progesterone plus FGF. Certain cultures contained AQ: Please check if the text “Some cultures contained…” needs rewording in terms of clarity.a dose actinomycin D (25µg/mL) to inhibit new RNA synthesis for 2, 4, and 6h (Rider *et al.*2003*b*). The stromal cells were collected in TRIzol and RNA was purified using the phase lock gel heavy tubes. The amount of *Wnt5a* mRNA was measured using semiquantitative PCR. The optical density of *Wnt5a* was divided by the optical density of *Gapdh* amplified from the same template. Three independent assays were performed.

### *Wnt5a* gene knockdown

Quiescent stromal cells were transfected with *Wnt5a* siRNA (5nM; Silencer Select) and non-targeting siRNA (5nM; Silencer Select) according to the manufacturer’s protocol using lipid-mediated transfection (Lipofectamine RNAiMAX Transfection Reagent; Ambion, Life Technologies Corp) at 48h of serum starvation. After 24h, the stromal cells were stimulated to synchronously enter the cell cycle by adding progesterone (1µM) and FGF (50ng/mL). The proliferative response was measured using the MTT assay. Results were obtained from three experiments with six replicate wells in each. 

Real-time PCR (Step-one; Applied Biosystems) was carried out according to the manufacturer’s protocol. Templates of *Wnt5a *were quantified using a Taqman probe and *Wnt5a *primers (Rn01402000; Applied Biosystems) specific for the *Wnt5a* gene. A Taqman probe and *Gapdh *(Hs99999905; Applied Biosystems)-specific gene primers were used for the internal control. The average Ct of *Gapdh* in quiescent stromal cells was 25.9±0.24, whereas the average Ct of *Gapdh* in stimulated stromal cells was 25.8±0.29, indicating no change in response to treatment. In each cycle, fluorescent signals for *Wnt5a* and *Gapdh* were collected from triplicate samples. The relative quantity (RQ value) was calculated and compared with cDNA synthesized from stromal cells stimulated to proliferate with progesterone plus FGF. Samples without template were included in triplicate on each plate as a negative control. 

### Statistical analysis

Differences between groups were analyzed using the nonparametric Mann–Whitney test. *P* values less than 0.05 (two-sided) were considered statistically significant.

## Results

### *Wnt5a* expression increases in response to progesterone pretreatment

**Figure 1:**
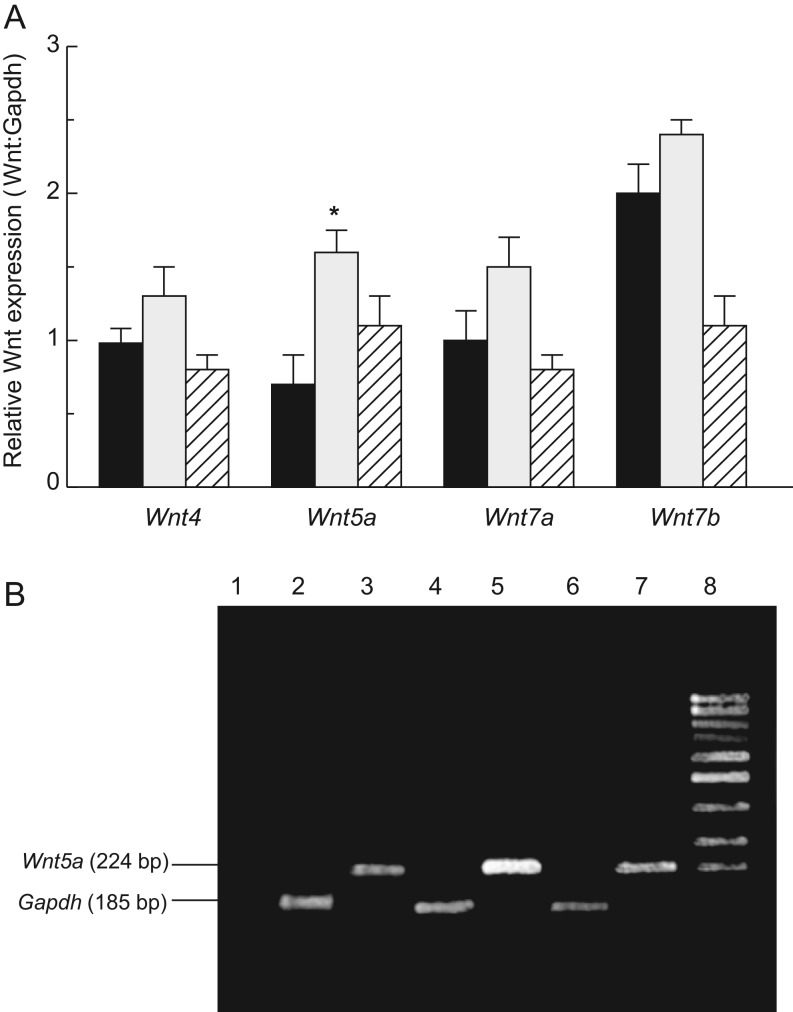
Progesterone increases *Wnt *transcript expression in rat uteri. RNA was isolated from OVX rat uteri, progesterone-pretreated uteri (0 hE), and progesterone-pretreated uteri followed by a single injection of estradiol (6 hE). The RNA was pooled from three rats at each treatment and converted into cDNA. *Wnt *target gene expression was compared with *Gapdh *in the same sample. (A) Quantitative assessment of the changes in the expression for four *Wnt* genes in response to progesterone pretreatment (0 hE) and progesterone pretreatment with a single injection of estradiol (6 hE). The *Wnt *target genes were compared with *Gapdh* in the same sample. Data are mean values ± s.e.m. from four replicates for each treatment group. Black boxes are data from OVX uteri, gray boxes are data from progesterone-pretreated uteri (0 hE), and stippled boxes are data from progesterone-pretreated uteri given a single injection of estradiol (6 hE). *P<.05, Mann–Whitney U test. (B) A representative gel of the PCR products using the *Wnt5a* target gene primers and *Gapdh* gene primers. Lane 1, no template, negative control; lane 2, OVX uteri with *Gapdh *primers; Lane 3, OVX uteri with *Wnt5a *primers; lane 4; 0hE with *Gapdh* primers; lane 5, 0hE with *Wnt5a *primers; lane 6, 6hE with *Gapdh *primers; lane 7, 6 hE with *Wnt5a* primers; lane 8, molecular size markers.

In order to determine the *Wnt *family members involved in stimulating uterine stromal cell proliferation and differentiation, expression of candidate *Wnt* transcripts were measured in rat uterine RNA using semiquantitative PCR (Fig. 1A). We compared *Wnt* expression in the uterine RNA isolated from OVX rats, those pretreated with progesterone (72h progesterone, 0 hE), and those pretreated with progesterone and estradiol for 6 h (6hE) to stimulate stromal cell re-entry into the cell cycle. Of the four *Wnt *genes studied, expression of all increased numerically in response to progesterone alone, and then declined partially with the addition of estradiol. The magnitude of change was significantly greater (*P*<0.05) for *Wnt5a *in response to progesterone compared with the amount in OVX rat uteri. A representative gel of PCR products from rat uteri shows the increase in *Wnt5a* transcripts in response to progesterone alone (Fig. 1B).

### Changes in the spatial distribution of Wnt proteins in response to steroid hormones

**Figure 2:**
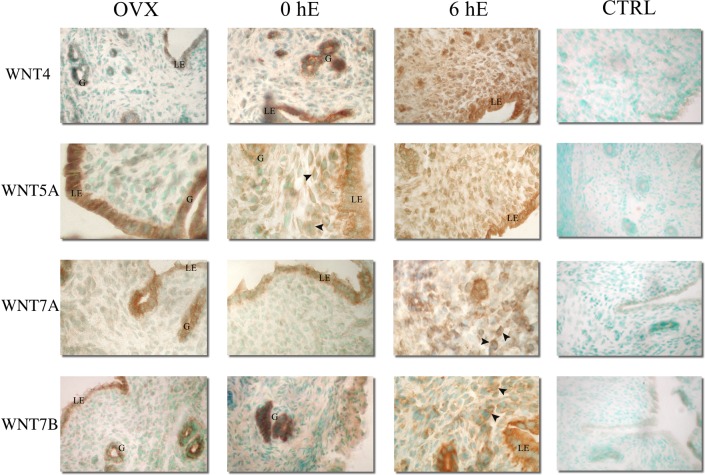
The spatial distribution of WNT proteins changes in response to sex steroids. Paraffin-embedded rat uteri were sectioned as detailed in the text and the cell-specific distribution of WNT proteins was assessed using standard immunocytochemistry. Ovariectomized=OVX, 0 hE=progesterone pretreated, 6hE=progesterone pretreated plus estradiol for 6 h, CTRL, control sections counterstained with methyl green. LE, luminal epithelium; G, glandular epithelium. Arrowheads indicate positive cells in the stroma. Original magnifications: 400 and 1000×.

The cell-specific distribution of the four WNT proteins was compared among rat uteri in the absence of hormone (OVX), progesterone pretreatment (0 hE), and progesterone pretreatment followed by estradiol exposure for 6 h (6 hE). In OVX rat uteri, WNT4 protein was evident in the luminal and glandular epithelia and that cell-specific distribution remained after rats were treated with progesterone (Fig. 2, 0hE). Progesterone pretreatment followed by 6h of estradiol stimulated WNT4 expression in the uterine stromal cells and expression was maintained in the epithelial cells (Fig. 2, 6hE). WNT5A protein was expressed in the luminal and glandular epithelial cells of OVX rat uteri (Fig. 2). In response to progesterone pretreatment, WNT5A protein was evident particularly in the periluminal uterine stromal cells as shown by arrowheads in Fig. 2, 0hE. Expression was maintained in the epithelium. In response to progesterone pretreatment followed by estradiol administration, WNT5A was expressed in the epithelial and stromal cells (Fig. 2, 6hE). WNT7A was expressed in the epithelial cells of OVX rat uteri (Fig. 2, OVX). The cell-specific expression did not change in response to progesterone pretreatment (Fig. 2, 0hE). However, in response to progesterone pretreatment followed by exposure to estradiol, WNT7A was detected in some stromal cells as well as the epithelial cells (Fig. 2, 6hE). WNT7B was expressed in the luminal and glandular epithelial cells of uteri from OVX rats (Fig. 2, OVX). Progesterone pretreatment did not affect the cellular distribution of WNT7B protein (Fig. 2, 0hE). However, the glandular expression was strong in progesterone-pretreated uteri (0hE). In uteri from rats pretreated with progesterone and estradiol, WNT7B was detected in epithelial cells and in some stromal cells (Fig. 2, 6hE).

Taken together, the results indicated that progesterone pretreatment not only enhanced *Wnt5a* transcript expression but that WNT5A protein was evident in the periluminal stromal cells, which proliferate and differentiate into decidual cells. 

### Progesterone increases WNT5a expression in proliferating uterine stromal cell lines

**Figure 3:**
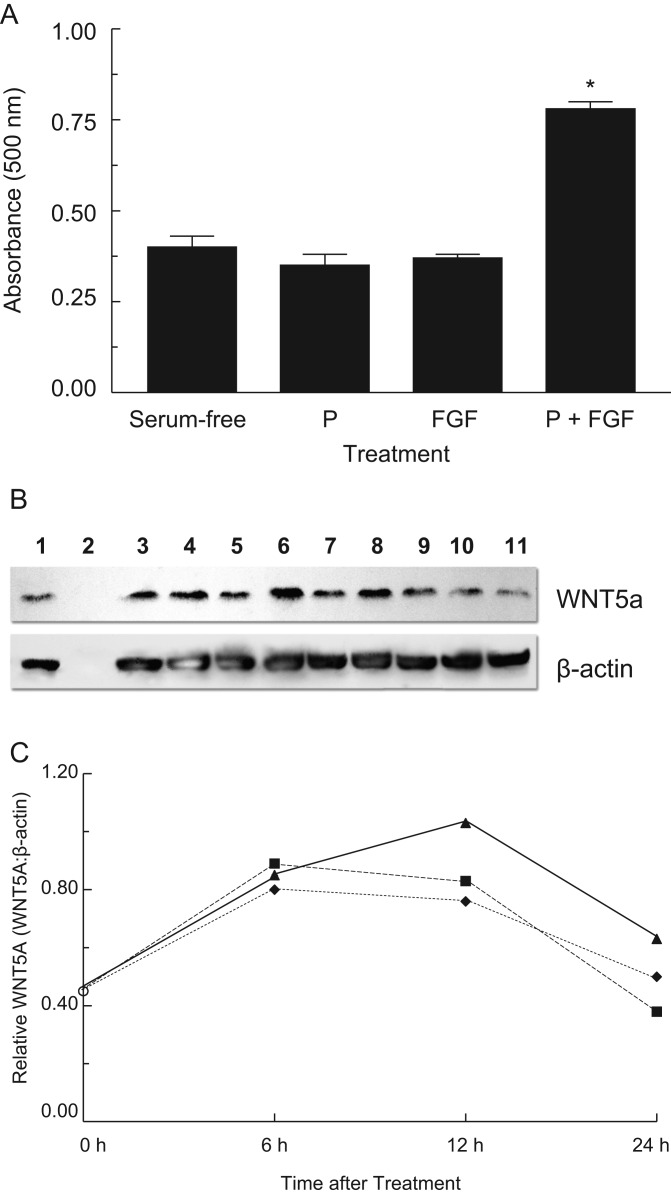
Progesterone plus FGF increase proliferation and WNT5A expression in uterine stromal cell lines in culture. (A) Quiescent uterine stromal cells (UIII, passage 20) were stimulated with progesterone, FGF, and progesterone plus FGF. The proliferative response was measured 48 h after stimulation using the MTT reagent. Data are mean values ± s.e.m. from three experiments with three replicates in each experiment. *P<0.05, Mann–Whitney U test. (B) Proliferating stromal cells were collected at various time points after stimulation, and the expression of WNT5A protein was measured by scanning densitometry of western blots. Representative western blot of WNT5A expression in uterine stromal cells stimulated with progesterone, FGF, and progesterone plus FGF. Lane 1, proteins from rat uteri treated with progesterone for 3 days (positive control); lane 2, no extract (negative control); lane 3, uterine stromal cells treated with progesterone for 6 h; lane 4, uterine stromal cells treated with FGF for 6 h; lane 5, uterine stromal cells treated with progesterone plus FGF for 6 h; lane 6, uterine stromal cells treated with progesterone for 12 h; lane 7, uterine stromal cells treated with FGF for 12 h; lane 8, uterine stromal cells treated with progesterone plus FGF for 12 h; lane 9, uterine stromal cells treated with progesterone for 24 h; lane 10, uterine stromal cells treated with FGF for 24 h; lane 11, uterine stromal cells treated with progesterone plus FGF for 24 h. (C) Quantitative changes in WNT5A expression in uterine stromal cells stimulated with proliferative agents. The amount of WNT5A was adjusted to β-actin in the same sample. WNT5A numerically increased in response to progesterone (triangle), FGF (diamond), and progesterone plus FGF (box) with approximately a two-fold increase at 6 and 12 h in response to progesterone alone. Data are mean values from two independent experiments.

To extend this observation and gain further insight into the consequence of progesterone regulation of WNT5A expression, a well-characterized rat uterine stromal cell line was utilized. Cultured uterine stromal cells (UIII) were stimulated with progesterone (1µM) and FGF (50ng/mL) to induce proliferation. Proliferation increased significantly (*P*<0.05) as expected (Fig. 3A) in response to progesterone plus FGF. No increase in cell number was detected in stromal cells stimulated with progesterone alone or FGF alone.

To determine whether WNT5A was a progesterone target in proliferating stromal cells, total RNA and proteins were sequentially isolated from stromal cells cultured with progesterone and FGF at various times (0, 6, 12, and 24h). WNT5A was detected by western blotting in all treatment groups (Fig. 3B). WNT5A increased approximately two-fold in all stromal cells at 6h post stimulation (Fig. 3C). WNT5A protein was numerically maximal in response to progesterone (triangle) at 12h post progesterone treatment. At 12h post stimulation with progesterone plus FGF (diamond) and FGF (box ) alone, the amount of WNT5A declined slightly from the amount measured at 6h post stimulation.

**Figure 4:**
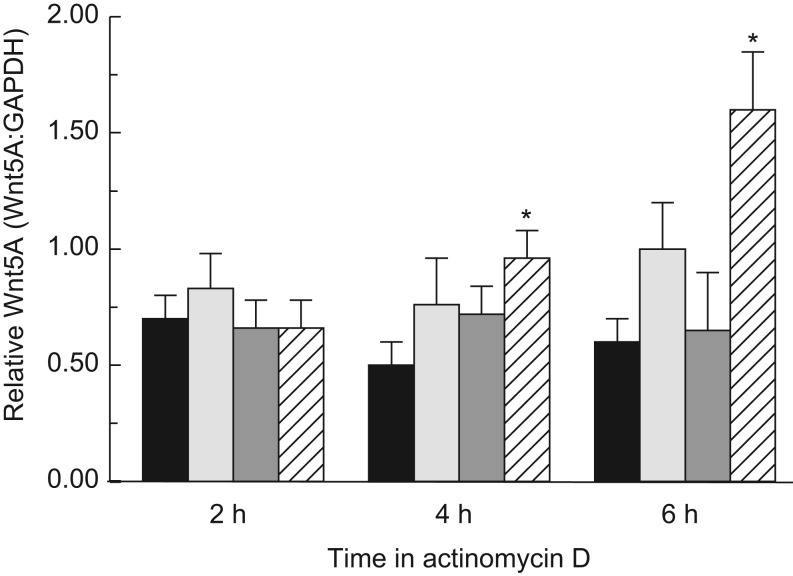
Progesterone plus FGF stabilize *Wnt5a* mRNA. Quiescent uterine stromal cell lines (UIII, passage 23) were stimulated with progesterone, FGF, and progesterone plus FGF in medium containing actinomycin D to inhibit new mRNA synthesis. The amount of *Wnt5a* mRNA was measured using semiquantitative PCR as detailed in the text. Data are mean values ± s.e.m. from three independent experiments. Black boxes, serum-free medium; white boxes, progesterone alone; gray boxes, FGF alone; stippled boxes progesterone plus FGF. *P<0.05, Mann–Whitney U test.

Surprisingly, semiquantitative PCR analysis of *Wnt5a* transcripts after treatment of cells with proliferative agents (progesterone plus FGF) revealed no changes in the amount of *Wnt5a* mRNA at these same time points (data not shown). To investigate whether progesterone and FGF stabilized *Wnt5a* mRNA, uterine stromal cells were stimulated with progesterone alone, FGF alone, and progesterone plus FGF in the presence of actinomycin D to inhibit new mRNA synthesis (Fig. 4). The relative amount of *Wnt5a* transcripts increased numerically in response to progesterone alone after 6h of inhibition. Progesterone plus FGF increased (*P*<0.05) *Wnt5a* transcripts at 4 and 6h of culture in actinomycin D. Together, the results suggest that progesterone plus FGF increased *Wnt5a* expression, in part, by stabilizing its mRNA.

### WNT5a is essential for stromal cell proliferation 

**Figure 5:**
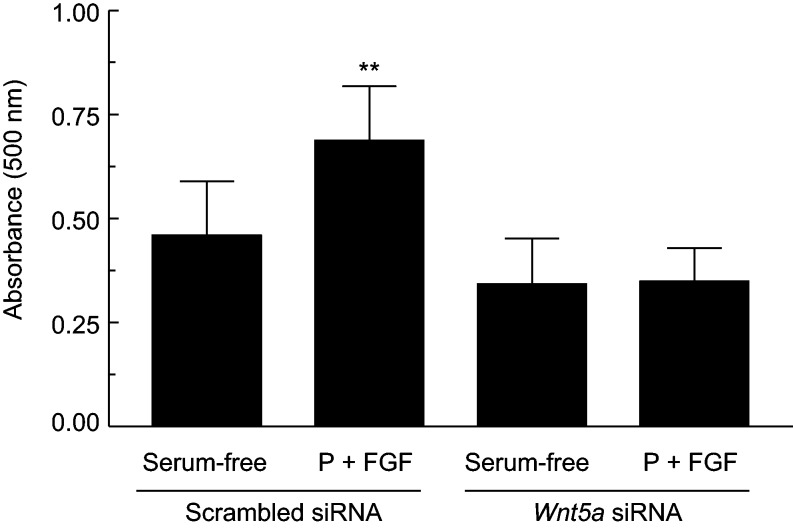
Inhibition of *Wnt5a* blocks progesterone-induced stromal cell proliferation. Quiescent uterine stromal cells (UIII) were transfected with siRNA 24 h before stimulation. Transfected stromal cells were stimulated with progesterone plus FGF to induce proliferation. The cells were collected at 48 h after addition of mitogenic agents. Proliferation was assessed using the MTT assay. Progesterone plus FGF stimulated stromal cell proliferation significantly in stromal cells transfected with scrambled siRNA but not with *Wnt5a *siRNA. **P<0.004, Mann–Whitney U test. Data are mean values ± s.d. of three independent assays with six replicates in each experiment.

In response to progesterone and estradiol, uterine stromal cells proliferate and differentiate into the decidua. Previous studies from our laboratory (Piva *et al.* 1996) and the current results (Fig. 3A) indicate that progesterone plus FGF stimulate stromal cell lines to proliferate. To test whether WNT5A was a target of progesterone-mediated stromal cell proliferation, stromal cells were stimulated with progesterone and FGF (Fig. 5). Some cells were transfected with scrambled siRNA, whereas other cells received *Wnt5a*-specific siRNA. The proliferative response was assessed using the MTT assay (Fig. 5). Progesterone plus FGF increased stromal cell number in the presence of scrambled siRNA (*P*<0.004, Mann–Whitney U test). For cells transfected with *Wnt5a* siRNA, there was no effect of progesterone plus FGF on cell number. Real-time PCR revealed *Wnt5a *mRNA only in stimulated stromal cells transfected with scrambled siRNA. *Wnt5a* mRNA was not detected in quiescent (serum-free) stromal cells or in cells stimulated after transfection with *Wnt5a* siRNA.

## Discussion

The purpose of this study is to gain additional insight into the signaling mechanisms regulating the progesterone-dependent switch in proliferation from epithelium to stroma. Attention was focused on uterine stromal cells because these cells differentiate into the decidua and interface with the fetal placenta. All of the four genes studied exhibited an increase in transcript expression in response to progesterone alone. The expression of WNT5A protein in the endometrium was restricted to the epithelial cells before progesterone treatment. Progesterone treatment specifically induced WNT5A in the uterine stromal cells. To investigate the mechanism(s) underlying the proliferative switch from epithelium to stroma, well-characterized uterine stromal cell lines were utilized. Progesterone plus FGF stimulated stromal cell proliferation. Proliferation was accompanied by increased WNT5A protein but not mRNA. Inhibition of new mRNA synthesis in stromal cells stimulated to proliferate revealed an increase in *Wnt5a* transcripts in response to progesterone that was further enhanced by progesterone plus FGF. These results indicate that progesterone plus FGF increase WNT5A expression in proliferating uterine stromal cells in part by stabilizing *Wnt5a* mRNA. Moreover, knockdown of WNT5A blocked stromal cell proliferation, indicating the importance of WNT5A signaling in regulating the uterine stromal cell cycle.

Stromal cell proliferation and differentiation are required to alter the uterus to a receptive state for embryo implantation (Dey *et al.* 2004, Ramathal *et al*. 2010). Proliferation and differentiation are largely under the control of female sex steroids, estradiol and progesterone, acting through their cognate receptors (Lydon *et al*. 1995, Franco *et al*. 2012, Pawar *et al.* 2014, Thouas *et al.* 2015). Evidence suggests that these hormones act in a temporal and spatially ordered fashion regulating cell–cell communication between epithelial and stromal compartments and through paracrine actions within compartments. In rodents, estradiol stimulates proliferation of uterine epithelial cells (Martin & Finn 1968). At day 4 of pregnancy in the rat, proliferation is redirected to stromal cells by progesterone inhibition of epithelial cell proliferation (Rider & Psychoyos 1994) and down-regulation of stromal cell GSK-3β (Rider *et al.* 2006). Administration of the progesterone receptor antagonist, RU486, at days 1 and 2 *post-coitum* stimulated epithelial cell proliferation in rat uteri; however, the progesterone-dependent proliferative switch to stromal cells was blocked (Rider & Psychoyos 1994). More recently, tissue-specific knockout of the progesterone receptor in the epithelial cells of mouse uteri allowed for the continued proliferation of epithelial cells in the absence of progesterone receptor action (Franco *et al.* 2012). In contrast to the RU486 blockade of progesterone receptor action in both compartments, however, the proliferative switch to stroma occurred normally in the absence of epithelial progesterone receptors, suggesting that progesterone-dependent proliferation in the stroma is controlled by stromal not epithelial cell progesterone receptors. 

The canonical WNT5A signaling pathway stabilizes β-catenin. As β-catenin accumulates, it translocates to the nucleus and activates cell cycle regulators such as cyclin D and c-myc (Zimmerman *et al*. 2012). Progesterone stimulates the expression of WNT5A and the accumulation of β-catenin in rat uterine stromal cells consistent with canonical WNT5A signaling. Previously, we found that progesterone pretreatment of rat uteri increases the expression of cyclins D1 and D3, both *Wnt *signaling targets (Rider *et al.* 2006). The current results are consistent with the idea that WNT5A, acting through the canonical pathway, is an early and essential signal in the progesterone-dependent proliferative switch from epithelia to stroma because blockade of this pathway inhibits stromal cell proliferation. 

Non-canonical WNT5A signaling regulates cell adhesion and cell movement and provides positional information to cells (Katoh & Katoh 2007, Endo *et al.* 2015). *Wnt* signaling facilitates changes in the cytoskeleton and junctional complexes among cells (Zimmerman *et al*. 2012). It is interesting to note that conditional deletion of the *Cx34* (*Gjb4*) gap junction protein gene does not prevent stromal cells from proliferating; however, the cells do not differentiate (Laws *et al.* 2008). The results from this study show an essential role for WNT5A in stromal cell proliferation. Other studies (Cha *et al.* 2014) suggest that WNT5A acts through non-canonical signal transduction to promote decidualization. It is possible that non-canonical signaling shuts off the progesterone-β-catenin-TCF pathway and activates polar cell migration (Minami* et al.* 2010) necessary for decidualization. It is now important to determine whether stromal cell proliferation and differentiation are coupled through WNT5A; however, the signaling pathway for proliferation is different than that for differentiation. 

There is ample evidence that epithelial–stromal cross talk is essential during the initial implantation reaction (Mericskay *et al*. 2004, Nallasamy *et al.* 2012). The spatial distribution of the four *Wnt* genes in OVX rat uteri revealed hormone-independent expression in the glandular and luminal epithelia. WNT7B expression appeared to increase in the glandular epithelia in response to progesterone alone; however, the fold-change in expression was not measured. In the adult mouse uterus, WNT7B expression increases in response to estradiol (Hayashi *et al.* 2009). The function of *Wnt7b* in the adult uterus, however, is not known. 

Progesterone plus estradiol decreased transcript expression of the four *Wnt *genes studied. The spatial distribution of WNT proteins, however, changed in response to estradiol, and all four WNT proteins were expressed in the progesterone-pretreated stromal cells in response to estradiol. It is notable that WNT5A was the only protein expressed in the progesterone-pretreated stromal cells. Of additional interest was the strong expression of WNT4 in the stromal cells of rat uteri following 6h of estradiol injection. Culture of primary human endometrial cells with cyclic AMP and steroid hormones stimulate their differentiation as assessed by expression of the decidual cell markers prolactin and IGFBP (Li *et al*. 2013). WNT4/β-catenin signaling was essential for differentiation as blockade of either WNT4 or β-catenin reduced the expression of decidual cell markers. The increased expression of several WNT proteins in the stroma in response to progesterone and estradiol suggests that a number of different *Wnt* genes are important for uterine stromal cell differentiation. Our results suggest that *Wnt5a* is an early signaling event in the stroma that is essential for stromal cell proliferation. Understanding the spatial and temporal activation of *Wnt *signaling in response to sex steroids will improve understanding of endocrine control of cell proliferation and differentiation.

## Declaration of interest

The authors declare that there is no conflict of interest that could be perceived as prejudicing the impartiality of the research reported.

## Funding

The research was supported in part by the NIH (National Center for Research Resources (SP20RR016475) and the National Institute of General Medical Sciences (8P20GM103418)). 

## Author contribution statement

V R wrote the paper, trained the students, and designed the experiments. A T and Z K carried out the *Wnt *localization and expression studies. A B conducted the siRNA experiments. S F and J W are responsible for the western blot analyses and PCR analyses of *Wnt5a* in cultured stromal cells. B K conducted the statistical analysis and participated in the experimental design. All authors have reviewed this manuscript before its submission.
